# Uncertainty of leukoencephalopathies: a case report

**DOI:** 10.1186/s13256-021-03089-6

**Published:** 2021-11-16

**Authors:** Mohamed A. Taha, T. Scott Diesing

**Affiliations:** Department of Neurological Sciences, University of Nebraska Medical Center, Omaha, NE USA

**Keywords:** Adult-onset leukoencephalopathies, White matter lesions, Rapidly progressive dementia, Medical uncertainty

## Abstract

**Background:**

Adult-onset leukoencephalopathies are a group of heterogeneous disorders characterized by white matter abnormalities. Leukoencephalopathy is usually encountered in children, but here we report a case with adult-onset leukoencephalopathy. Also, we explore this concept of uncertainty in medicine by discussing the approach to this case that has multiple possible etiologies.

**Case presentation:**

A 70-year-old Caucasian male presented with a subacute onset of cognitive impairment and mood disturbances associated with behavioral changes. Neuroimaging demonstrated high-intensity lesions involving cerebral white matter. Progressive dementia and cognitive decline followed. Multiple possible etiologies are discussed based on the patient presentation and risk factors.

**Conclusion:**

Adult-onset leukoencephalopathy can become a diagnostic challenge. Certain approaches need to be developed to explore the uncertainty of such conditions and to improve diagnostic yield.

## Introduction

Leukoencephalopathies are a group of disorders that affect adults and are characterized by the development of white matter changes seen on imaging. The differential for leukoencephalopathy is broad and can be a diagnostic challenge. In many cases, the diagnosis of leukoencephalopathy requires a brain biopsy to confirm the diagnosis, which is an invasive procedure.

## Case presentation

A 70-year-old right-handed Caucasian male was brought by his family for an evaluation of progressive memory loss and change in his behavior over the last month. He had not had any prior neurologic or psychiatric illnesses. He became socially withdrawn, and subsequently became unable to independently perform activities of daily living. His speech output lessened to the point that he would speak only in single words. Up to the time of presentation, there had been no motor or movement concerns, hallucinations, incontinence, or fevers.

His history was notable for well-controlled hypertension and hyperlipidemia. He had been diagnosed with rheumatoid arthritis 20 years prior and was treated with long-term oral methotrexate (15 mg per week) and occasional use of diclofenac. He was a former smoker (stopped smoking for 30 years) and a social drinker without history of illicit drug use. His family medical history was noncontributory.

Physical examination

His affect was flat, and he had impaired attention and concentration. He was unable to follow simple commands. There were no detectable abnormalities on ocular or cranial nerve examinations. Strength was intact throughout, but tone was increased in both lower extremities. Coordination examination was notable for bilateral upper extremity action tremor, and ataxia of gait. Hyperreflexia was noted in both biceps and knee reflexes. Palmomental reflex was present and symmetric. The examination was notably absent of clonus, myoclonus, or excessive movements. His presentation was reminiscent of a partially akinetic-mute state.

Investigations

**Fig. 1:**
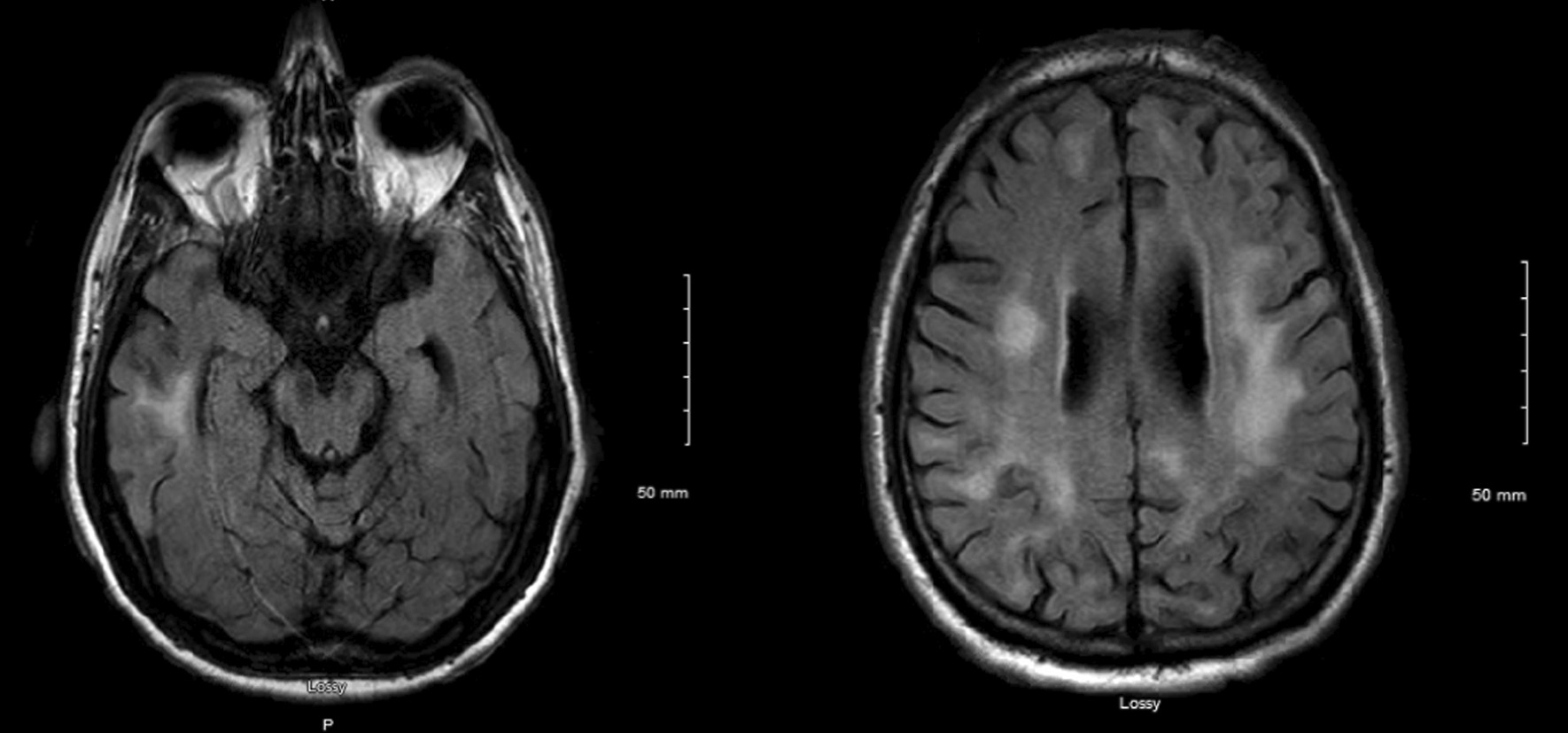
FLAIR signal brain MRI showing extensive subcortical white matter hyperintense lesions

Test results are presented below (Tables 1, 2). Cerebrospinal fluid (CSF) analysis showed elevated protein content with normal white blood cell counts. Special CSF testing showed elevated levels of 14-3-3 protein. Real-time quaking-induced conversion (RT-QuIC) was negative. Autoimmune and paraneoplastic studies were negative. Electroencephalography performed over several days on multiple occasions showed diffuse background theta slowing without epileptiform discharges or electrographic seizures. Magnetic resonance imaging (MRI) brain with and without contrast showed extensive patchy subcortical white matter lesions on T2 and fluid-attenuated inversion recovery (FLAIR) sequences (Fig. 1). MRI also showed mild diffuse volume loss without parenchymal or meningeal enhancement. CT angiography of the head and neck was unremarkable. CT of the chest, abdomen, and pelvis showed mild mesenteric and hilar lymphadenopathy. Subsequent mesenteric lymph node biopsy was unremarkable.

**Table 1:** Lists laboratory tests and their results

Test	Result	Test	Result
CSF protein	94 (15–45)	Albumin, CSF	Elevated
CSF glucose	77 (40–70)	T-tau protein, CSF	1973 pg/mL
CSF WBCs	1	Arylsulfatase A WBC	Within normal limits
CSF RBCs	< 3000	Phytanic acid	Within normal limits
CSF albumin	43 (14–26)	HgA1C	5.3%
CSF IgG	4 (0–6)	Hilar lymphadenopathy biopsy	Negative for neoplasm
Serum albumin	4 (3–4)	Acetaminophen and salicylate levels	Within normal limits
CSF ACE	2 (0–2)	CSF T-tau protein	1900
PET scan	Small hilar lymphadenopathy	CSF 14-3-3 protein	Positive

*CSF*, Cerebrospinal fluid; *WBCs*, White blood cells; *RBCs*, Red blood cells; *ACE*, Angiotensin converting enzyme; *HgA1C*, HemoglobinA1C

**Table 2:** List of negative laboratory tests

CSF microbiology tests
HIV serology
CSF Lyme antibody
CSF *Tropheryma whipplei* PCR
CSF RT-QuIC
CSF encephalopathy panel
CSF flow cytometry
CSF JCV DNA (repeated three times)
CSF VDRL
CSF bacterial and fungal cultures
CSF West Nile IgM/IgG
UDS
CBC
Urine heavy metals
Serum ceruloplasmin

*RT-QuIC*, Real-time quaking-induced conversion; *JCV*, John Cunningham virus; *VDRL*, Venereal disease research laboratory; *UDS*, Urine drug screen; *CBC*, Complete blood count

The patient’s cognitive impairment and akinetic mutism continued to deteriorate concurrent with development of urinary incontinence. A 3-day course of high-dose intravenous methylprednisolone (1000 mg each day) was empirically given without any clinical improvement. A selective serotonin reuptake inhibitor was tried without success. Two cycles of electroconvulsive therapy (ECT) were not effective. Unfortunately, the patient died a month after presentation from complications of aspiration pneumonia.

This clinical presentation is ambiguous. Progressive cognitive impairment was notably involved with memory, behavior, and mood disturbances along with late-onset urinary incontinence. Upper motor neuron signs and spasticity were detected in the lower extremities with mild ataxia. We will discuss the differential diagnoses of this ambiguous presentation.

Exploring the uncertainty

Progressive multifocal leukoencephalopathy (PML) is a multifocal, white matter disease that occurs in immunosuppressed patients (Table 3). It is manifested by altered mental status, cortical symptoms (aphasia, motor weakness), and gait ataxia [1]. On neuroimaging, PML can appear as symmetrical or asymmetrical, single or multifocal white matter lesions. Sometimes it can also involve gray cortical matter [2]. The diagnosis is made by demonstrating a positive PCR of John Cunningham (JC) virus DNA with or without brain biopsy. JC virus DNA in CSF has a sensitivity of 92% [3]. PML with negative CSF JC virus PCR can also be seen in patients with human immunodeficiency virus (HIV) [3]. The PML diagnosis is possible in this case as the clinical presentation and MRI findings are suggestive. However, it remains uncertain as CSF was negative for JC virus in two separate samples.

**Table 3:** Summary of the clinical features of a selected leukoencephalopathy group

Leukodystrophy	Clinical and diagnostic features
Vanishing white matter disease	Cognitive decline, ataxia, and seizures; MRI showing confluent FLAIR signal abnormalities and global atrophy
Metachromatic leukodystrophy	Progressive cognitive decline, UMN signs, dystonia and behavioral changes; MRI showing symmetrical periventricular white matter changes; diagnosis by arylsulfatase A enzyme levels
Krabbe disease	Spastic paraplegia, dystonia sensory symptoms, and ataxia; MRI showing white matter lesions predominately affecting parieto-occipital and cerebellar areas; globoid cells found on biopsy; decrease in galactocerebrosidase levels
Adrenoleukodystrophy	Progressive spastic paraplegia, urinary and sensory symptoms; MRI showing variable brain white matter changes and spinal myelopathy features; increase in very-long-chain fatty acid levels
Alexander disease	Motor and ataxia symptoms sometimes associated with bulbar, dementia, and behavioral changes; MRI showing white matter lesions predominantly affecting frontal region
Myelinoclastic diffuse sclerosis (Schilder’s disease)	Acute inflammatory demyelinating white matter disease associated with increased intracranial pressure and tumor-like presentation

*UMN*, Upper motor neuron; *MRI*, Magnetic resonance imaging; *FLAIR*, Fluid attenuated inversion recovery

*Creutzfeldt–Jakob disease (CJD)* is a prion disease that is characterized by subacute progressive dementia associated behavioral and motor impairments [4]. Cognitive decline involves areas of high cortical function such as aphasia, apraxia, and frontal lobe function. Behavioral changes include apathy, emotional lability, and anxiety. All these features were found in our patient. Also, myoclonus can be seen in CJD. Spasticity and extensor plantar responses are found in CJD, which were detected in our patient. Akinetic mutism is also one of the features of late-stage CJD. MRI findings commonly seen are diffusion-weighted imaging (DWI) and FLAIR hyperintensity signals in putamen and caudate nucleus. Sometimes white matter changes can be detected [5]. The findings of our case were inconsistent with the commonly seen MRI findings in CJD as MRI showed extensive white matter changes only. Moreover, the progression of MRI findings of cases of CJD was not seen with the repeat MRI in our case. Electroencephalography (EEE) in this case did not show the characteristic periodic spike wave complexes that can be seen in 65% of cases with CJD [6]. Real-time quaking-induced conversion (RT-QuIC) sensitivity ranges from 92% to 95% in cases of CJD [7]. The negative RT-QuIC makes the diagnosis of CJD unlikely. On the other hand, the specificity of 14-3-3 is around 80%, that is, there are high rates of false positives [8]. CSF 14-3-3 can be positive in other cases such as paraneoplastic encephalopathies and metabolic encephalopathy. In conclusion, the clinical features were suggestive of CJD, although neuroimaging and CSF testing made the diagnosis unlikely.

Adult-onset leukoencephalopathies are a heterogeneous group of white matter diseases that affect myelination and are manifested by dementia and psychiatric and movement symptoms. Usually they present early in life, but adult-onset leukodystrophies can be seen [9–11]. Features of leukodystrophies with similar clinical presentation are summarized in Table 3. None of the adult-onset leukoencephalopathies fully explains the clinical picture of this patient. In addition, enzyme levels were not affected.

Autoimmune and paraneoplastic encephalitis have a wide clinical presentation ranging from limbic encephalitis to neuropsychiatric symptoms [12]. FLAIR lesions can affect both white and gray matter, unlike the MRI findings in this case. Antibody panel that screens for autoimmune and paraneoplastic encephalitis was negative. A whole-body scan was unremarkable except for mild hilar and mesenteric lymphadenopathy, which had not shown any neoplastic features on histological assessment. Lastly, the lack of clinical response to a trial dose of intravenous steroids makes this diagnosis unlikely.

Primary central nervous system (CNS) lymphoma is a rare neoplastic disorder that presents with change in behavior, mood abnormality (depression), and sometimes focal neurological deficits and seizures [13]. Primary CNS lymphoma appears as either hypointense or isointense on T2 MRI sequence and contrast homogeneously [14]. This is different from what was seen in this patient. CSF analysis with two cytology screenings was negative for lymphoma cells. All these findings make this diagnosis unlikely.

Finally, methotrexate (MTX)-induced neurotoxicity has been described in multiple cases. It can be divided into acute, subacute, and chronic [15]. Aseptic meningitis can be seen in the acute setting after receiving MTX. It presents with features of meningitis, including fever, vomiting, and headache, and is generally transient after stopping MTX [15]. Leukoencephalopathy can be detected after long-term use of MRX and is usually mild and symptomatic and can disappear after therapy cessation. On the contrary, disseminated necrotizing leukoencephalopathy (DNL) is a rare, fatal form of methotrexate-induced neurotoxicity [16]. DNL usually occurs with intrathecal and intravenous MTX. However, DNL was also reported in patients on low-dose oral MTX [17]. It can happen months after the initiation of treatment. Clinically, DNL presents with changes in personality, progressive dementia, and sometimes seizures and motor symptoms. It can lead to death in a few months. Brain MRI usually shows extensive subcortical white matter lesions (patchy or confluent) that appear on T2 and FLAIR signals. Sometimes, these lesions can give some contrast enhancement. White matter changes can affect all brain regions, especially the posterior region (occipito-parieto-temporal), and can thus be mistaken for posterior reversible leukoencephalopathy (as initially in this patient), which can also be seen in patients on methotrexate. Finally, DNL is progressive and does not respond to cessation of treatment. The features of DNL do resemble the clinical presentation of this case; however, it is difficult to confirm the diagnosis in the absence of a brain biopsy.

## Discussion

The case reviewed exemplifies the uncertainty that physicians face in their daily practice. Some cases can be simple and straightforward, while others are more complex and ambiguous. Thus, it is it is important for physicians to be comfortable with the concept of uncertainty, a concept that is rarely discussed in the medical literature.

Leukoencephalopathies are a group of disorders that affect adults and are characterized by the development of white matter changes seen on imaging. The differential for leukoencephalopathy is broad and can be a diagnostic challenge. In many cases, the diagnosis of leukoencephalopathy requires a brain biopsy to confirm the diagnosis, which is an invasive procedure. In this case, the patient’s long-term use of oral methotrexate suppressed his immune system and made him susceptible to opportunistic infections. JC-virus-related leukoencephalopathy is one of the major white matter diseases that can be seen in immunosuppressed patients, and it can be confirmed by demonstrating the presence of JC virus DNA in CSF. However, there are an increasing number of patients who can be diagnosed with JC-negative PML [3]. The clinical presentation of this patient, which can be summarized as progressive dementia with behavioral changes, akinetic mutism, and upper motor neuron signs, can be explained by PML, but the diagnosis is difficult to make given his negative JC virus in CSF. Other etiologies that can present with similar symptoms and signs along with patchy leukoencephalopathy on MRI include adult-onset leukoencephalopathies such as Krabbe, Alexander disease, and metachromatic leukoencephalopathies. However, as mentioned previously, their neuroimaging features differ from this patient, and they are also rare diagnoses. Disseminated necrotizing leukoencephalopathy is the most plausible explanation for this patient’s presentation and is consistent with the MRI findings. However, brain biopsy is needed to confirm the diagnosis and to exclude other causes of leukoencephalopathies. In summary, the approach of white matter disease in adults should involve a multidisciplinary approach that includes a collection of detailed history and risk factors, and the evaluation of subtle clinical signs in addition to gathering laboratory results. Despite that, a brain biopsy will often be needed.

## Conclusion

The principle of minimum uncertainty states that, in the case of alternative solutions to a problem, we should accept only the solutions in set solutions that provide the minimal amount of uncertainty [18]. In our medicine practice, we face uncertainty almost on a daily basis. While a diagnosis may not be certain, physicians need to reduce the amount of uncertainty to the smallest solution set, or differential. The diagnostic process of leukoencephalopathy in adults can be complex and requires basic knowledge of processing uncertainty.

## Data Availability

The datasets used and/or analyzed during the current study are available from the corresponding author on reasonable request.
